# Turkish Hazelnut Extracts Exert Anti-Proliferative and Anti-Cancer Effects on Colorectal Cancer HCT-116 Cells

**DOI:** 10.3390/cimb48010001

**Published:** 2025-12-19

**Authors:** Banu Bayram, Evren Demircan, Atefeh Karimidastjerd, Elvan Yılmaz Akyüz, Yusuf Tutar

**Affiliations:** 1 Department of Nutrition and Dietetics, Faculty of Health Sciences, University of Health Sciences, Istanbul 34668, Türkiye; banu.bayram@sbu.edu.tr (B.B.); elvan.yilmazakyuz@sbu.edu.tr (E.Y.A.); 2 Department of Food Engineering, Faculty of Chemical and Metallurgical Engineering, Istanbul Technical University, Istanbul 34469, Türkiye; evrendemircan@itu.edu.tr; 3 Department of Food Engineering, Faculty of Chemical and Metallurgical Engineering, Yıldız Technical University, Istanbul 34210, Türkiye; atefeh.karimi64@gmail.com; 4 Department of Basic Medical Sciences, Division of Biochemistry, Faculty of Medicine, Recep Tayyip Erdoğan University, Rize 53100, Türkiye; 5 Molecular Oncology Program, Health Sciences Institute, Recep Tayyip Erdogan University, Rize 53100, Türkiye; 6 Molecular Medicine Program, Health Sciences Institute, Recep Tayyip Erdogan University, Rize 53100, Türkiye; 7 Rize Training and Research Hospital, Recep Tayyip Erdogan University, Rize 53020, Türkiye

**Keywords:** anticancer, apoptosis, colorectal cancer, hazelnut extract, mitochondrial membrane potential

## Abstract

The rising incidence of cancer has demanded the development of new anti-cancer chemical sources. The presence of phenolics in hazelnut cell cultures has led to the development of new and potential pharmacotherapeutic uses. Hazelnut extract has emerged as a promising candidate due to its high phytochemical content. HCT-116 colorectal cancer IC_50_ cell viability of Palaz and Tombul hazelnut extracts was determined as 400 μg/mL and 200 μg/mL, respectively. Flow cytometry annexin V-fluorescein isothiocyante (FITC) apoptosis detection indicated apoptosis of Tombul hazelnut extract and Palaz hazelnut extract as 23.53% and 17.47%, respectively. The apoptosis result of flow cytometry was also supported at the protein level. Hazelnut extracts resulted in an increased loss of MMP as well. The loss of MMP has significantly increased from an average of 0.61% to 16.17% in Tombul hazelnut extract and to 20.38% in Palaz hazelnut extract. This is further supported by screening *MICU1, MICU2, PPAR-γ, PPARGC1A, UCP1, UCP2*, and *UCP3* gene expressions. Targeting apoptosis pathways, particularly MMP, is an effective strategy for cancer prevention and treatment. Hazelnut extract contains phenolic compounds, which activate these pathways, resulting in enhanced apoptosis in colorectal cancer cells. The phenolic contents of Palaz and Tombul hazelnut extracts were determined as 271.72 ± 5.3 mg gallic acid equivalent (GAE)/100 g sample dry weight (DW) and 85.23 ± 2.2 mg GAE/100 g sample DW, respectively. Further, hazelnut extract may reduce oxidative stress, contributing to its anti-cancer properties. The extracts could be utilized as functional ingredients in foods and nutraceuticals to assist with cancer prevention and treatment.

## 1. Introduction

Cancer is a disorder that is responsible for one in six deaths worldwide. In 2020 cancer caused approximately 10 million deaths. It is predicted that there will be a 77% increase in the number of new cancer cases in 2050 as compared to cancer cases in 2022 that accounts for 35 million new cancer cases [1]. Cancer prevention is an important research area. Surgery, chemotherapy, and radiation therapy are the most prevalent treatment options for cancer. However, the safety risks and serious adverse effects forced researchers to seek new methods [2,3]. The development of safe, novel, and effective drugs with less toxicity and less side effects is under investigation for cancer therapeutics.

The relationship between dietary components, especially phytochemicals, and cancer risk has gained significant attention in recent years, particularly with respect to the potential health benefits of various foods. Phytochemicals may control cancer progression in different ways including upregulation of tumor suppressor genes, inhibition of oncogenes, showing antioxidant and immunomodulatory activities, promoting DNA repair, regulating hormones, suppressing pathways involved in promoting cancer and cancer progression, modulating epigenetic mechanisms, and causing cell death through apoptosis [4,5]. Fruits, vegetables, herbs, nuts, and medicinal plants are sources of phytochemicals exerting anti-cancer effects that can be extracted from different parts of plants including roots, leaves, seeds, peels and shells. For example, sulforaphane, is a well-known anti-cancer compound found in cruciferous vegetables that enhances the expression of pro-apoptotic genes, modulates cell cycle, and induces apoptosis [6,7]. Curcumin in turmeric is a strong immunomodulatory compound that regulates tumor development by promoting apoptosis via a p53-dependent mechanism [8] and PI3K/Akt signaling pathway [9]. Aronia leaf extracts that are rich in anthocyanins downregulated the migration and invasion of SW-480 and HT-29 cells as a model of colorectal cancer (CRC) [10]. Among the sources of phytochemicals, hazelnuts (*Corylus avellana* L.) have emerged as a subject of interest due to their rich nutritional profile, which includes high levels of antioxidants, dietary fiber, and bioactive compounds such as phenolic acids, flavonoids, and tocopherols, which are known for their antioxidant properties. As oxidative stress is a known contributor to cancer development, antioxidants play a crucial role in mitigating this risk [11,12]. Hazelnuts may also influence cancer risk through their fatty acid composition. The modulation of lipid metabolism is crucial as certain fatty acids can affect cell signaling pathways related to cancer progression [13].

CRC is the third leading cancer type in the world. In 2020 the number of new cases of colon and rectum cancer were 1.93 million. The interplay between hazelnuts and cancer is complex and multifaceted, involving antioxidant properties, dietary fiber content [14], modulation of gut microbiota [15,16], and the presence of phytochemicals that target cancer stem cells [17,18]. These components are believed to play a crucial role in modulating cancer risk, particularly in the context of CRC. Compounds with high antioxidant activity of hazelnut derived extracts (skin, oil, shell) could play a role in reducing oxidative stress that is associated with CRC [19,20,21].

It is widely accepted that targeting apoptosis pathways is an effective strategy for cancer prevention and treatment. One of the key players in the regulation of apoptosis is the mitochondrion, which serves as a central hub for apoptotic signaling. The mitochondrial membrane potential (MMP) is a critical factor in this process, as it influences the release of pro-apoptotic factors, thereby activating the caspase cascade that leads to cell death. The relationship between MMP and apoptosis has been extensively studied, revealing that a decrease in MMP is often an early event in the apoptotic process [22,23,24]. The interplay between MMP and apoptosis is a critical area of research with significant implications for health and disease. Both biochemical and molecular tools are essential to revealing this mechanism.

This study aims to investigate the cytotoxic effects and mitochondrial functions of Turkish hazelnut extracts on HCT-116 human colorectal carcinoma cell lines, as well as their effect on the proliferation and apoptosis of the cells, then reveal the inhibitory mechanism against the cell growth. As CRC is the third leading cancer type in the world and there is a lack of studies investigating the effects of Turkish hazelnut extracts on CRC cells, we focused on this cancer type. In addition, the expression of seven important genes involved in the regulation of apoptosis and MMP were analyzed by evaluating their mRNA levels in the HCT-116 cell line.

## 2. Materials and Methods

### 2.1. Chemicals and Reagents

Dulbecco’s Modified Eagle Medium High Glucose (DMEM) and SybrGreen Master Mix was obtained from EuroClone (Pero, Italy). Dimethyl sulfoxide (DMSO), phosphate-buffered saline (PBS), fetal bovine serum (FBS), MTT (3-(4,5-Dimethylthiazol-2-yl)-2,5-Diphenyl Tetrazolium Bromide), penicillin/streptomycin, Trypsin-EDTA, methanol, formic acid, Folin–Ciocalteu phenol reagent, hexane, acetone, and sodium carbonate were purchased from Sigma-Aldrich (Darmstadt, Germany). As phenolic standards, gallic acid, protocatechuic acid, catechin, 4-hydroxybenzoic acid, caffeine, chlorogenic acid, vanillic acid, epigallocatechin gallate, caffeic acid, syringic acid, vanillin, p-coumaric acid, ferulic acid, sinapic acid, ethyl 3,4 dihydroxybenzoate, resveratrol, rutin, phloridzin, myricetin, t-cinnamic acid, naringenin, pinobanksin, quercetin, hesperetin, luteolin, kaempferol, apigenin, isorhamnetin, rhamnetin, and pinostrobin were used. All standard compounds were purchased from Sigma-Aldrich (Darmstadt, Germany). ApopNexin Annexin-V-FITC with PI Apoptosis Kit was obtained from Merck (Darmstadt, Germany). Total RNA Isolation Kit and SensiFast cDNA Synthesis Kit were purchased from Analytik Jena (Jena, Germany), and Meridian Bioscience (Cincinati, OH, USA), respectively. Ultra-pure water was produced using water purification system Elga Purelab Flex 3 (Elga Labwater, Woodridge, IL, USA) to prepare solvent mixtures containing water.

### 2.2. Sample Collection and Phenolic Extract Preparation

Hazelnut samples used in the study were collected from Ünye, Ordu, Türkiye in 2022. Tombul and Palaz varieties were selected for this study as they are unique to Türkiye and they are known for their high phenolic content, as reported in the literature [25,26] which promises their anti-cancer activity. In addition, Tombul variety has been predominantly cultivated in Türkiye (30%).

Samples were preserved in cool bags for transportation to the laboratory. Hazelnuts were extracted using acetone/water (80:20; *v*/*v*) to obtain phenolics with higher yields as compared to other solvents, as referred to in the literature [27,28,29,30]. Hazelnuts were shelled and finely ground with their skins using a mixer. To remove the oil, 50 mL hexane was added to 7.5 g of ground hazelnuts, homogenized in an ultraturrax device for 2 min, and then centrifuged for 3 min at 3500 rpm. The hexane phase was discarded, and this step was repeated twice. The resulting residue was placed in Petri dishes, and the hexane was removed under a fume hood. A total of 50 mL 80% acetone was added to the residue and incubated for 30 min in a 50 °C water bath for extraction. At the end of incubation, the extracts were centrifuged for 10 min at 3500 rpm. This step was repeated twice. The collected liquid fractions were combined and concentrated with a rotary evaporator (Büchi Rotavapor II, Büchi, Flawil, Switzerland). The hazelnut extract was frozen in a lyophilizer. Extracts were dissolved in methanol/water (80:20; *v*/*v*) filtered through a 0.45 µm membrane filter.

### 2.3. Total Phenolic Content

Total phenolic content was determined using the Folin–Ciocalteau method specified in the study of Singleton and Rossi [31]. An amount of 1 ml of 10% 0.2 N Folin–Ciocalteu reagent was added to hazelnut extracts. The volume of 0.75 mL (6%) saturated sodium carbonate solution was added to this mixture and mixed. The sample was kept in the dark for 90 min and then measured at 765 nm in a spectrophotometer (Synergy HT; BioTek Instruments, Winooski, VT, USA). Total phenolic substance content was given as gallic acid equivalent using the gallic acid in the calibration curve.

### 2.4. HPLC Analysis of Hazelnut Phenolics

HPLC analyses were conducted with Shimadzu 20A Series liquid chromatograph (Shimadzu Corporation, Kyoto, Japan). It was a complete system with a microvacuum degasser, autosampler, column oven, controller, PDA detector, and LC Solution software Version 1.25 SP5. The HPLC protocol that was used in this study was validated by Gültekin-Özgüven et al. [32] and used in different articles [33,34]. The chromatogram detection was conducted between wavelengths of 190 and 600 nm. In the screening, the standards containing protocatechuic acid, catechin, 4-hydroxybenzoic acid, chlorogenic acid, vanillic acid, gallic acid, epigallocatechin gallate, quercetin, hesperetin, caffeic acid, luteolin, syringic acid, p-coumaric acid, ferulic acid, sinapic acid, ethyl 3,4 dihydroxybenzoate, resveratrol, rutin, phloridzin, myricetin, t-cinnamic acid, naringenin, pinobanksin, kaempferol, apigenin, isorhamnetin, rhamnetin, and pinostrobin were analyzed. ACE C18 column (250 mm × 4.6 mm, 3 μm) was used to separate the phenolics. Water with 0.75% formic acid (*v*/*v*) (solvent A) and HPLC-grade methanol with 0.75% formic acid (*v*/*v*) (solvent B) were used for a gradient of mobile phase. The flow rate was 0.5 mL, the injection volume was 10 μL, and the column temperature was 40 °C. The total duration of the gradient program was 64 min. The gradient started with 5% solvent B and was maintained for 6 min. It reached 40% B at 12 min, 80% B at 48 min, and 100% B at 51 min. It decreased to 5% B at 58 min. The phenolics present in the samples were identified and quantified by comparing the retention time and the size of the peaks in the extracts with those of the standards.

### 2.5. Cell Culture

HCT-116 cells were obtained from ATCC (Catalog number—CCL 247). DMEM supplemented with 10% FBS and 1% penicillin/streptomycin was used for cell culture. Flasks of 25 cm^3^ were used for the seeding and incubated at 37 °C in a 5% CO_2_ atmosphere. After 80–90% confluence of cells, Trypsin-EDTA solution (0.25% trypsin and 0.03% EDTA) was used to detach cells [35].

### 2.6. Cell Viability Assay

Cell viability was determined by the MTT (3-(4,5-dimethylthiazol-2-yl)-2,5-diphenyltetrazolium bromide) method. The cells were seeded in 96-well plates at a density of 5 × 10^3^ per well. At the end of the 24 h incubation period, the medium was removed and freshly prepared medium containing hazelnut extracts (1–5000 μg/mL) was added. Viability test was performed for 24 and 48 h. After incubation, the medium was replaced with 100 μL of fresh medium containing 1 mg/mL MTT dissolved in PBS. Cells were incubated in the dark at 37 °C for 3 h. Then, the medium was discarded and 100 μL DMSO was added to each well. The absorbance was measured at 570 nm with a microplate reader (Thermo Scientific, Multiskan Go, Vantaa, Finland). Viability was expressed using the ratio of the absorbance of treated sample to untreated sample multiplied by 100. DMSO was used a negative control [36]. MTT analysis was used to determine the IC_50_ dose.

### 2.7. Annexin V Apoptosis Analysis

Annexin V-FITC apoptosis Detection Kit with PI, ApopNexin^TM^FITC (Merck, Germany) was used to determine apoptosis. HCT-116 cells were seeded in 6-well plates at the density of 1 × 10^6^ cells. After 24 h, the hazelnut extracts were applied, and at the end of 48 h of incubation, the cells were washed twice with 5 mL of cold phosphate buffer. The cells were removed with Trypsin-EDTA and centrifuged for 5 min at 400 rpm. Each of the cells obtained was suspended with 1 mL of cold 1× Binding Buffer (Sigma, St. Louis, MO, USA). A total of 200 μL of the mixture was used to conduct flow cytometry analysis. ApopNexin^TM^ FITC (3 μL) and 100× propidium iodide (2 μL, Sigma, St. Louis, MO, USA) were added to the solution for the analysis and mixed. After 15 min incubation time in the dark at room temperature, the samples were measured using CytoFLEX flow cytometry (Beckman Coulter, Brea, CA, USA). The excitation wavelength was set as 488 nm and the emission wavelength was set as 530 nm. DMSO was used as control [37].

### 2.8. Apoptotic Marker Expression at Gene and Protein Level at HCT-116 Cancer Cells

Commercially available Bax, Bcl-2, and Bcl-xL human ELISA kits were used to quantify HCT-116 cell lysates. The cells were treated with Tombul and Palaz extracts, and the cells were harvested and lysed in RIPA buffer. Manufacturer protocols were applied to quantify apoptosis proteins. While Bax protein level increased, Bcl-2 protein level decreased and the ratio of Bax/Bcl-2 and Bax/Bcl-xL ratios increased. These changes indicate enhanced activation of the intrinsic apoptosis pathway (Mybiosource Human Elisa Kit, San Diego, CA, USA). Primers of apoptotic pathway genes were obtained from Tutar lab in-house cancer PCR array (desalted and HPLC grade from Merck) GAPDH employed for constitutive expression and normalization [38].

### 2.9. Determination of Mitochondrial Membrane Potential

A Mitochondrial Membrane Potential Mito^PT^ TMRE kit (Biorad, Feldkirchen, Germany) was used to analyze the MMP following the process steps given by the manufacturer. HCT-116 cells were seeded at a concentration of 3 × 10^5^ cells/well. Forty-eight hours later, positive control (carbonyl cyanide m-chlorophenylhydrazone, CCCP) and negative controls (DMSO) were created for 60 min after the analysis. At the end of incubation, cells were trypsinized and pellets were collected by centrifugation at 400 rpm for 5 min. After the pellets were collected, cells were washed with PBS. Pellets were collected by centrifugation again at 400 rpm for 5 min. One mL of 200 nM Mito^PT^ dissolved in 1× working buffer was added and the cells were suspended then incubated in the incubator at 37 °C for 15 min [39]. After incubation, cells were analyzed by CytoFLEX flow cytometry (Beckman Coulter, Brea, CA, USA).

### 2.10. RNA Isolation and cDNA Synthesis

HCT-116 cells were collected and cultured in 6-well plates at a density of 3 × 10^5^ cells/well. InnuPREP RNA mini kit 2.0 (Analytik Jena, Germany) was used for RNA isolation. The kit used is based on the Trizol method. The concentration of RNA was measured by absorbance at 260 nm and RNA purity was determined by A260/A280 ratio. The isolated RNAs were kept at −20 °C until qRT-PCR analysis. In this study, cDNA Reverse Transcription kit (SensiFAST^TM^ cDNA Synthesis Kit, Bioline GmbH, Luckenwalde, Germany) was used for cDNA synthesis. cDNA synthesis of *MICU1, MICU2, PPAR-γ, PPARGC1A, UCP1, UCP2*, and *UCP3* genes was performed with a Biorad device. Obtained cDNAs were stored at −20 °C until RT-PCR. Primer pairs were prepared by performing BLAST+ 2.13.0. The designed primers were synthesized by a commercial company. The primer sequences of the examined genes are given in Table 1. A total of 100 μmol/μL of the primers were made by dissolving the stock with nuclease-free sterile water. Amplifications were carried out with region-specific forward and reverse primers and cDNA using SYBRGreen Master Mix in a total reaction volume of 20 μL (Primer; 1 μL, SYBR green master mix; 10 μL, water; 8 μL, cDNA; 1 μL). The cDNA obtained using an Analytical Jena qRT-PCR device (Analytical Jena, Germany) was subjected to qRT-PCR analysis [36]. OneTaq^®^2X Master Mix with Standard Buffer kit (New England Biolabs, Ipswich, MA, USA) was used in the qRT-PCR experiment to amplify DNA samples with the selected primers. For normalizing the results *GAPDH* was used as the housekeeping gene. Gene expression rates were calculated using the 2^−Δ^^ΔCt^ method for relative expression analysis and results were given as fold change differences.

### 2.11. Statistical Analysis

Data were presented as mean ± SD. Number of replicates was three (n = 3). Statistical analyses were evaluated by Graph Pad Prism 5.0 (San Diego, CA, USA). The significance of differences in experimental from control was assessed by Student’s *t* test or a one-way ANOVA. The statistically significant difference between different treatment groups was indicated as *p* < 0.05.

## 3. Results

### 3.1. Total Phenolic Content of Hazelnut Extracts and Determination of Individual Phenolic Compounds

The total phenolic content of hazelnut extracts was determined by using a spectrophotometric method. Hazelnuts are rich in phenolic compounds contributing to their anti-cancer activity. According to the results, the phenolic content of Palaz and Tombul hazelnut extracts were determined as 271.72 ± 5.3 mg GAE/100 g sample DW and 85.23 ± 2.2 mg GAE/100 g sample DW, respectively. The range of total phenolic content values in this study is much greater than Özcan et al. (2018), Jakopic et al. (2011), and Kalkan et al. (2015) who found 46–63 mg/100 g, 8.7–47.8 mg/100 g phenolics, and 74 mg/100 g phenolics in hazelnut kernel, respectively [40,41,42]. Also, Del Rio et al. (2011) found lower phenolic content in hazelnut skin (4.1–12.7 mg/100 g) of different cultivars belonging to Türkiye, Italy, and Chile [43].

**Figure 1:**
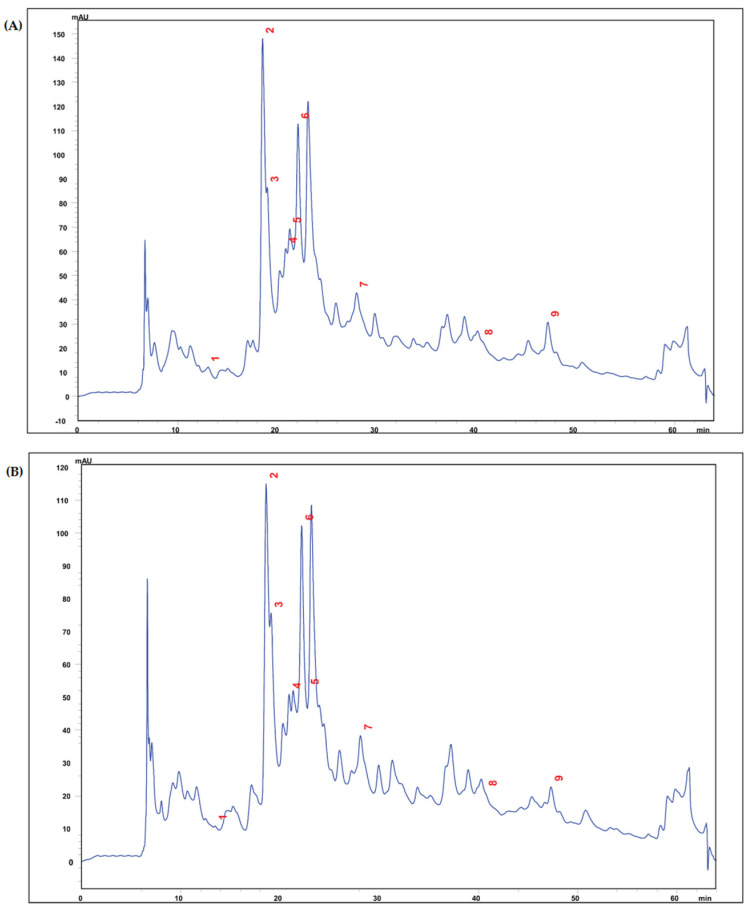
HPLC chromatograms of Tombul (**A**) and Palaz (**B**) hazelnut kernel extracts with UV detection at 280 nm. (1) gallic acid (RT: 13.4 min), (2) protocatechuic acid (RT: 18.5 min), (3) vanillic acid (RT: 19.0 min), (4) catechin (RT: 20.9 min), (5) epigallocatechin gallate (RT: 21.3 min), (6) epicatechin (RT: 22.1 min), (7) syringic acid (RT: 28.1 min), (8) t-cinnamic acid (RT: 40.6 min), and (9) p-coumaric acid (RT: 47.3 min). RT; retention time.

In the present study, the hazelnut kernel extract in the mixture of methanol/H_2_O (80:20; *v*/*v*) was subjected to chromatography with PDA detector to identify the phenolics profile of different subclasses. A total of nine phenolic compounds were quantified in hazelnut extracts, and the concentration of phenolics differed among the cultivars (Table 2). HPLC identification of individual phenolics in the extracts of whole hazelnut kernels confirmed the presence of gallic acid, protocatechulic acid, vanillic acid, catechin, epigallocatechin gallate, epicatechin, syringic acid, t-cinnamic acid, and p-coumaric acid in all analyzed cultivars. Among the analyzed phenolics in the hazelnut kernels, epicatechin and protocatechulic acid were found as the most abundant phenolic compound varied between 8.28 and 9.89 mg/100 g sample DW and 4.05–5.18 mg/100 g sample DW, respectively. The identification of phenolic composition of hazelnuts corresponds with previously reported data on different hazelnut cultivars [43,44,45]. Representative chromatograms of Palaz and Tombul hazelnut extracts were shown in Figure 1.

### 3.2. Cell Cytotoxicity Assay

**Figure 2:**
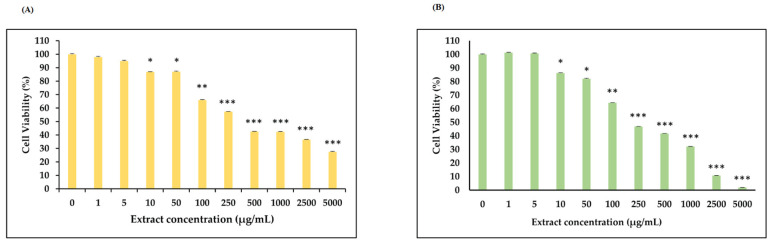
Cell viability analysis by MTT assay. (**A**) cell viability (%) of Palaz hazelnut extract treated (1–5000 μg/mL) on HCT-116 cells. IC_50_ value was 400 μg/mL. (**B**) cell viability (%) of Tombul hazelnut extract treated (1–5000 μg/mL) on HCT-116 cells. IC_50_ value was 200 μg/mL. Data are represented as mean ± SD, *** *p* < 0.001, ** *p* < 0.01, and * *p* < 0.05 compared with the control. IC_50_ value was 200 μg/mL.

The effect of hazelnut extracts on cell proliferation of HCT-116 cells was evaluated through MTT assay. The growth of hazelnut extract-treated cells was inhibited dose-dependently. Lyophilized hazelnut extracts were dissolved in DMSO and Palaz extract prepared at a concentration of 250 mg/mL and Tombul hazelnut extract prepared at a concentration of 200 mg/mL. In order to determine their effect on the viability of HCT-116 cells, the extracts were applied in the range of 1–5000 μg/mL to cells. While no toxic effect of hazelnut extracts was observed after 24 h, the result was obtained after 48 h. As a result of the MTT test, the IC_50_ value of Palaz and Tombul hazelnut extracts were determined as 400 μg/mL and 200 μg/mL, respectively, and these concentrations were applied in all analyses. The results of MTT assay were given in Figure 2.

### 3.3. Effect of Hazelnut Extracts on the Apoptosis

**Figure 3:**
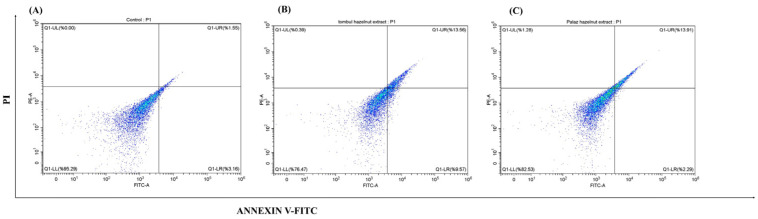
Representative dot plots of flow cytometric analysis of apoptosis with Annexin V-FITC/PI staining in hazelnut extract treated HCT-116 cells. Cells were treated with hazelnut extract at the IC_50_ dose. Cells were collected and subjected to Annexin V-FITC/PI staining and analyzed using flow cytometry. (**A**) Control, (**B**) Tombul hazelnut extract, (**C**) Palaz hazelnut extract. It was graphed as the means (±SD) of three independent experiments: LL indicates live cells; LR indicates early apoptosis; UR indicates late apoptosis; and UL indicates necrotic cells. Live cell populations in control, Tombul and Palaz were 95.29; 76.47; and 82.53, the total of early/late apoptosis cell populations were 4.71; 23.53; and 17.47 and necrosis cell populations were 0.00; 0.39; and 1.28, respectively. The results indicate that extracts drive HCT-116 cells to apoptosis.

**Figure 4:**
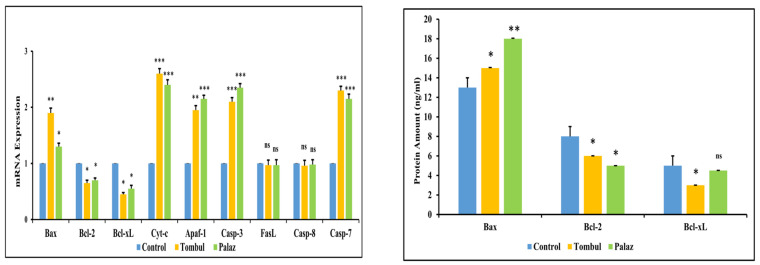
Extracts of Tombul and Palaz drive HCT-116 cells to apoptosis. mRNA expression of apoptotic pathway genes indicates intrinsic apoptosis pathway as evidenced by increased expression of pro-apoptotic genes (*Bax, Cyt-c, Apaf-1, Casp-3, Casp-7*) while antiapototic gene expressions (*Bcl-2, Bcl-xL*) were decreased. Extrinsic pathway gene expressions (*Casp-8* and *FasL*) remain unchanged (**Left**). Protein expressions of the HCT-116 cells treated with the extracts were screened by ELISA (**Right**). The results support mRNA expression and flow cytometry apoptosis results. * *p* < 0.05, ** *p* < 0.01, and *** *p* < 0.001 compared to the control group. "ns" refers to non-significant.

The effect of hazelnut extracts on HCT-116 cell apoptosis was investigated by Annexin V-FITC/PI staining method. Cell apoptosis was determined by flow cytometry/mRNA-Protein expression of apoptotic pathway markers, and the results were given in Figure 3 and Figure 4. As a result, it was observed that the live cell ratio decreased significantly from 95.29% to 76.47% with Tombul hazelnut extract and to 82.53% with Palaz hazelnut extract. Similarly, the proportion of late apoptotic cells increased significantly from 1.55% to 13.56% and 13.91% with Tombul and Palaz hazelnut extracts, respectively (*p* < 0.001). In contrast with the control group, the necrotic cell rates were 0.39% and 1.28% after treatment with Tombul and Palaz hazelnut extracts, respectively (*p* < 0.001). The result suggested that hazelnut extracts could induce HCT-116 cell apoptosis.

### 3.4. Effect of Hazelnut Extracts on Mitochondrial Membrane Potential

**Figure 5:**
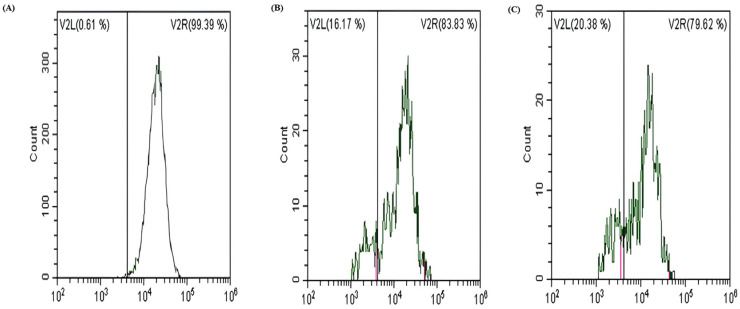
Mitochondrial membrane potential analysis by flow cytometry. (**A**) Control cells, (**B**) Tombul hazelnut extract-treated cells, (**C**) Palaz hazelnut extract-treated cells.

**Figure 6:**
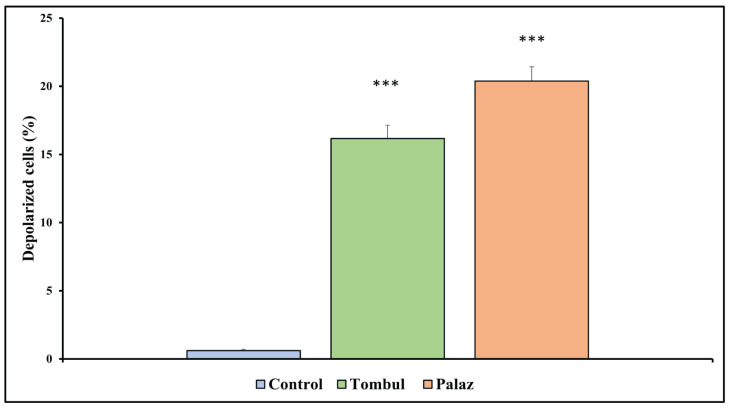
The percentage of depolarized cells Histogram changes in MMP in treated groups *** *p* < 0.001 compared to the control group.

The results of the MMP were shown in Figure 5 and Figure 6. Mitochondrial dysfunction is strongly related to apoptosis [46]. It has been stated that MMP collapse is a significant signal in early apoptosis [47,48]. The flow cytometry results demonstrated that the ratio of red/green fluorescence dramatically decreased in the hazelnut extract-treated cells (Figure 5), indicating that hazelnut extracts significantly reduced the MMP in HCT-116 cells, indicating mitochondrial dysfunction, and thus providing evidence that hazelnut extracts can induce apoptosis of tumor cells. As shown in Figure 5 and Figure 6, hazelnut extracts resulted in an increased loss of MMP. The MMP lost population has increased from an average of 0.61% to 16.17% in Tombul hazelnut extract and to 20.38% in Palaz hazelnut extract significantly (*p* < 0.001).

### 3.5. Effects of Hazelnut Extracts on Related mRNA Expression

The mRNA levels of MMP and apoptosis related genes including *MICU1, MICU2, PPAR-γ, PPARGC1A, UCP1, UCP2*, and *UCP3* were detected by RT-qPCR. Hazelnut extracts were applied at the IC_50_ dose to HCT-116 cells. Changes in the expression of genes were calculated according to the 2^−ΔΔCt^ method and the results were shown in Table 3. The mRNA expression levels of *MICU1, MICU2, PPAR-γ, UCP2*, and *UCP3* increased with the treatment of both Palaz and Tombul extracts. The mRNA expression level of *UCP1* was downregulated. No significant difference was observed for the *PPARGC1A* gene.

## 4. Discussion

A vast number of studies demonstrated that polyphenols can inhibit tumor generation, cell proliferation, and induce apoptosis in cancer cells [48,49,50,51,52]. Therefore, there has been a great interest in exploring natural compounds with anti-cancer effects from plants. The effects of numerous plant extracts on colon cancer cell lines have been studied, laying the groundwork for understanding the potential mechanisms of action and therapeutic applications [53,54,55]. The available literature on diverse natural extracts lays the groundwork for hazelnut extract’s impact on colon cancer cell lines and its ability to trigger apoptosis. Because of its high phytochemical content, hazelnut extract has emerged as a promising candidate, and we employed HCT-116 to screen the effect of phenolic-rich Palaz and Tombul hazelnut extracts. In our study, 80% acetone was used for phenolic extraction as it is a polar aprotic solvent that is widely used in the extraction of plant secondary metabolites, including phenolics. Its effectiveness is attributed to its ability to disrupt plant cell walls and solubilize a broad range of phytochemicals, which was reported in the literature as compared to other solvents [27,28,29,30]. It was found that Palaz and Tombul hazelnut extracts are rich in phenolic content (271.72 ± 5.3 mg GAE/100 g sample DW and 85.23 ± 2.2 mg GAE/100 g sample DW, for Palaz and Tombul hazelnut varieties, respectively). The effects of polyphenols found in extracts of different hazelnut parts have been widely researched in terms of cancer cell activity. However, there are a few articles in the literature dealing with the anti-cancer effect of Turkish hazelnut varieties. Tombul hazelnut leaf extract induced apoptosis in a dose-dependent manner and reduced migration capacity in lung and breast cancer [56]. In another study, roasted skinned Tombul hazelnut extract inhibited the growth of HT-29 cells by 89% [57]. Kalınkara cultivar hazelnut extract at 10 μM reduced the proliferation of human cancer cell lines including HeLa, MCF-7, MDA-MB-231, and A549 [58]. Additionally, the polyphenol-rich extracts from olive mill wastewater have chemopreventive properties, inhibiting colon cancer cell proliferation and migration [59]. This supports the concept that hazelnut extract, which contains similar bioactive chemicals, has similar effects on colon cancer cells via cellular activity. According to the reports, certain phenolic chemicals in plant extracts can have lethal effects on cancer cells. For example, a study on peanut phenolic compounds found that these compounds have histone deacetylase inhibitory activity, which is important in controlling gene expression associated with cancer cell proliferation. The study discovered that resveratrol, a phenolic molecule, has strong antiproliferative activity against colon cancer cell lines, implying that comparable chemicals in hazelnut extract could possibly contribute to its anti-cancer properties [60]. This shows that the bioactive components in hazelnut extract may suppress the development of colon cancer cells via a similar mechanism. Our phenolic content results for Palaz and Tombul hazelnut extracts are promising for anti-cancer treatment. Protocatechuic acid, vanillic acid, catechin, epicatechin, epigallocatechin gallate, and syringic acid were found to be the most abundant phenolics in both extracts. The hazelnuts were extracted with their skins. Hazelnuts and their by-products, such as skins, are considered important sources of phenolic compounds that exhibit strong antioxidant properties. The activity of this antioxidant is strongly associated with the phenolic content, recognized for aiding in the prevention of diseases related to oxidative stress, such as cancer [61]. The antioxidant capacity of hazelnut skins was over 100 times greater than that of hazelnuts lacking skins, emphasizing the major role of phenolic compounds in the antioxidant potential of hazelnut by-products [11]. The antioxidant phytochemicals found in hazelnuts, such as phenolic compounds, have been shown to scavenge free radicals and reduce oxidative stress, which is a key factor in the induction of apoptosis, thereby influencing apoptotic pathways [62,63]. The ability of these compounds to modulate apoptosis highlights the potential of hazelnuts as functional foods that can support cellular health. Although we had several unidentified peaks in the HPLC analysis, the total phenolic content of the extracts, as well as the amount of identified individual phenolic compounds were high as in accordance with the literature. The observed antiproliferative and anti-cancer effect of hazelnut extracts could be attributed to their phenolic content.

Along with their direct antioxidant properties, phenolic compounds might affect gene expression and enzyme activities tied to detoxification and regulation of the cell cycle. For instance, it was demonstrated that vanillic acid stimulates the expression of *Nrf2* and *HO-1*, key regulators of the cellular antioxidant response that have been associated with tumorigenesis suppression. The modulation of apoptotic pathways with vanillic acid also implies a mechanism through which this compound may prevent cancer cell survival [64]. Sari (2019) highlights that catechins can affect signal transduction pathways involved in cancer progression, such as those regulating cell cycle and apoptosis [65]. Furthermore, Yoshioka et al. (2022) [66] stated that catechins can regulate the ROS-mediated pathway and can lead to the induction of apoptosis in cancer cells. Additionally, catechins can increase the expression of tumor-suppressive microRNAs and decrease oncogenic microRNAs, thereby enhancing their anti-cancer effects [66]. In addition, catechins may inhibit the PI3K/Akt/mTOR pathway, which is frequently dysregulated in cancer. By targeting this pathway, catechins can suppress tumor growth, induce apoptosis, and enhance the sensitivity of cancer cells to chemotherapy [67]. As another abundant phenolic in our extracts, protocatechuic acid may induce apoptosis independent of ROS, as evidenced by the upregulation of pro-apoptotic protein and mRNA markers such as Caspase-3, p53, and Bax, and a significant reduction in anti-apoptotic marker Bcl-2 [68]. In addition, the ability of EGCG to induce apoptosis and promote cell cycle arrest has been demonstrated in a wide array of cancer cell lines and animal models. In vitro studies have shown that EGCG blocks carcinogenesis by affecting multiple signal transduction pathways, including JAK/STAT, MAPK, PI3K/AKT, and Wnt, thus affecting the transcription of tumor suppressor genes and protein synthesis [69]. In HepG2 hepatocellular carcinoma cells, EGCG has been shown to increase G0/G1 cell cycle arrest and stimulate apoptosis in a significant proportion of treated cells. Gene expression profiling revealed upregulation of autophagy inhibitory genes and apoptosis inducers, while several mitochondrial autophagy markers and autophagy regulator genes were downregulated [70].

Apart from phenolics, recent studies have identified hazelnut as a promising alternative source of taxol (Paclitaxel) and related taxane compounds. The anti-cancer efficacy of paclitaxel is well-documented, showing a broad-spectrum chemotherapeutic agent with proven activity against ovarian, lung, and breast cancers, among others [71]. Paclitaxel exerts its effects by stabilizing microtubules, leading to mitotic arrest and apoptosis in rapidly dividing cancer cells [60]. Although we did not analyze taxol in our study, this compound may play an important role in the results that we obtained.

Hazelnuts may also influence apoptosis through dietary components such as dietary fiber. It is well known that dietary fiber lowers oxidative stress and improves lipid metabolism. Due to their high dietary fiber content, hazelnuts may contribute to improved health outcomes by reducing oxidative stress [72]. Tuncil (2020) highlighted that dietary fiber rich hazelnuts are associated with improved bowel function and a reduced risk of colorectal cancer [14]. Hold (2016) emphasizes that the gut microbiota plays a critical role in the development of colorectal cancer and dietary choices can significantly influence the composition and function of the microbiota [73]. Healthier gut microbiota, in the production of short-chain fatty acids, may play a crucial role in cancer prevention [15], especially in reducing the risk of colorectal cancer.

The number of studies dealing with the effects of hazelnut on CRC cells is scarce. Therefore, in vitro cell culture studies using different cell types could be used to compare and support our results. Regarding the biological activities of hazelnut extracts, it was found that phenolic-rich hazelnut extracts induced apoptosis in HCT-116 cells as 23% and 17%, respectively. The apoptotic effects of numerous natural substances have been widely documented, with studies showing that chemicals such as resveratrol and kaempferol cause apoptosis in colon cancer cells by regulating apoptotic pathways [74]. Similarly, *Pleurotus eryngii* extracts have shown antiproliferative effects on colon cancer cell lines, resulting in considerable cell viability reductions [75]. The study found that at certain concentrations, the extract lowered the percentage of cell viability in the HCT-29 colon cancer cell line. This result is parallel to hazelnut extracts and displays a similar effect on colon cancer cell lines, causing apoptosis. Hazelnut extract contains comparable flavonoids, and activate these pathways, resulting in enhanced apoptosis in colon cancer cells. The antiproliferative properties of *Corylus avellana* cv. Kalınkara hazelnut cell culture extract were screened against the A549-lung cancer cell line [58]. Our results correlate with the literature reports as evidenced by the Palaz and Tombul hazelnut extracts. The studied extracts in this study displayed 17–23% apoptosis and the percentages are promising for potential use in the treatment of colorectal cancer.

Several studies on colon cancer have highlighted the importance of diverse natural extracts in modifying cell behavior via their effects on membrane potential. *Dictyopteris undulata* extract, for example, was shown to induce apoptosis in human colon cancer cells, as evidenced by mitochondrial membrane depolarization and the activation of apoptotic pathways [76]. In the study of Fuchs et al. (2020), among the phenolic extracts of winery by-products vine shoot extracts decreased MMP by 38% at a concentration of 200 μg/mL [77]. This implies that extracts from natural sources have a considerable impact on the membrane dynamics of cancer cells, potentially leading to cell death. The study shows polarization both by staining and screening MMP-related gene expressions (*MICU1, MICU2, PPAR-γ, PPARGC1A, UCP1,* *UCP2* and *UCP3*) and the extracts display enhanced membrane polarization, which leads to apoptosis.

We also investigated the expression levels of seven important genes in the context of apoptosis and MMP. *MICU1* and *MICU2* genes were significantly upregulated in hazelnut extract-treated cells (*p* < 0.001). The *MICU* gene is crucial for maintaining calcium homeostasis in mitochondria, thereby influencing various cellular processes, including apoptosis. The discussion surrounding *MICU* gene upregulation intersects with the broader implications of mitochondrial dynamics in health and disease. For instance, the study by Heo et al. (2009) indicates that mitochondrial dysfunction is a hallmark of various diseases, including neurodegenerative disorders, where the regulation of apoptosis is critically impaired [78]. This highlights the potential of targeting *MICU* and related pathways as therapeutic strategies to restore mitochondrial function and prevent cell death in pathological conditions.

*UCP1* is significantly downregulated, whereas *UCP2* and *UCP3* genes were significantly upregulated in hazelnut extract-treated cells. *UCP1* is a mitochondrial protein playing a critical role in thermogenesis. The studies have suggested that *UCP1* downregulation may be linked to apoptosis, particularly in the context of various pathological conditions. In the context of palmitic acid-induced podocyte apoptosis, Liu et al. (2018) demonstrated that mitochondrial dysfunction, characterized by decreased MMP, is a precursor to apoptosis [79]. This suggests that *UCP1* downregulation could contribute to mitochondrial dysfunction, thereby facilitating the apoptotic process. The authors suggest that the downregulation of *UCP1* in these conditions may enhance the susceptibility of cells to apoptosis, linking metabolic dysregulation to increased cell death [80]. The potential therapeutic implications of targeting *UCP1* in apoptosis-related conditions are noteworthy. For example, the study by Ocker and Höpfner (2012) emphasizes the importance of understanding apoptotic pathways in cancer therapy, suggesting that modulating *UCP1* expression could enhance the efficacy of treatments that induce apoptosis in cancer cells [81]. Conversely, *UCP2* is upregulated in our study. This can either promote cell survival by reducing oxidative stress or facilitate apoptosis by altering mitochondrial dynamics. For example, elevated *UCP2* expression has been associated with enhanced cell proliferation and resistance to apoptosis in cancer [82]. Moreover, the regulation of mitochondrial dynamics by *UCP2* is critical in the context of programmed cell death. Research has shown that *UCP2* influences mitochondrial permeability transition, a process that can lead to cytochrome c release and the activation of apoptotic pathways [83]. This duality underscores the importance of context in determining the outcomes of *UCP2* upregulation.

*PPAR-γ* gene was significantly upregulated in our hazelnut extract-treated cells. It is a nuclear receptor that plays a crucial role in regulating cellular metabolism, inflammation, and apoptosis. *PPAR-γ* activation can influence the expression of genes involved in mitochondrial biogenesis and dynamics, thereby affecting MMP and apoptosis [84]. It has been shown that targeting *PPAR-γ* modulates mitochondrial function and induces apoptosis in several studies [85,86] supporting our study.

The *PPARGC1A* gene is known for its involvement in mitochondrial biogenesis and energy metabolism. In our study no change in the expression levels of the *PPARGC1A* gene was observed in treatment groups. Accordingly, studies have shown that mitochondrial dysfunction and the subsequent loss of MMP can occur independently of *PPARGC1A* expression levels. For instance, in the context of neurodegenerative diseases, mitochondrial dysfunction has been linked to increased susceptibility to apoptosis, regardless of the expression of genes like *PPARGC1A* [87]. The relationship between *PPARGC1A* and apoptosis is further complicated by the findings that certain apoptotic stimuli can lead to mitochondrial dysfunction even in the presence of normal *PPARGC1A* levels. For example, exposure to toxic compounds such as Irgarol 1051 has been shown to induce apoptosis in HepG2 cells through mitochondrial dysfunction and oxidative stress, indicating that the apoptotic process can be activated through pathways that may not directly involve *PPARGC1A* [88].

## 5. Conclusions

The combination of findings from multiple studies underlines the promising function of natural extracts in cancer therapy, underlining the necessity for additional research in this field. The current study investigated phenolic-rich hazelnut extracts for their potential anti-cancer property on the HTC-116 cell line. According to the results of flow cytometry, both extracts drove cancer cells to apoptosis. Also, hazelnut extracts increased mRNA expression of pro-apoptotic genes while decreasing anti-apoptotic gene expressions. Furthermore, hazelnut extracts increased the loss of MMP. The results obtained from gene expression analysis of MMP related genes have further supported this data. The evidence suggests that polyphenol rich hazelnut extract may have a considerable influence on colon cancer cell line. The processes underlying this could include membrane potential reduction, apoptosis, and antioxidant bioactive compounds lowering oxidative stress. The intersection of hazelnuts and apoptosis presents a promising area of research that warrants further exploration. The antioxidant properties of hazelnuts, their potential to influence apoptotic pathways, and their role in modulating oxidative stress highlight their importance in health and disease. As research continues to explore the intricate relationships between diet and cancer, hazelnuts may play a significant role in dietary strategies aimed at cancer prevention. Both extracts could be utilized as a functional ingredient in foods and nutraceuticals, to assist with cancer prevention and treatment. The integration of hazelnuts into a balanced diet, particularly within the framework of the Mediterranean diet, may not only provide direct health benefits but also promote overall well-being, thereby contributing to cancer prevention strategies. Further research is warranted to identify the particular pathways and molecular targets implicated in hazelnut extract’s anti-cancer properties, which could lead to its potential application as a supplementary medicine in colon cancer treatment.

## Figures and Tables

**Table 1:** Sequences of forward and reverse primers used in Q-PCR.

Gene	Primer Sequences 5′→3′
*MICU1*	F: GACAGTGGCTAAAGTGGAGCTC
	R: CCTCTCATCAGCCGTTGCTTCA
*MICU2*	F: GGATGGCAGTTTTACAGTCTCCG
	R: GAAGAGGAAGTCTCGTGGTGTC
*UCP1*	F: AGTTCCTCACCGCAGGGAAAGA
	R: GTAGCGAGGTTTGATTCCGTGG
*UCP2*	F: TGGTCGGAGATACCAAAGCACC
	R: GCTCAGCACAGTTGACAATGGC
*UCP3*	F: GACTATGGACGCCTACAGAACC
	R: CTCCTTGAGGATGTCGTAGGTC
*PPAR-γ*	F: AGCCTGCGAAAGCCTTTTGGTG
	R: GGCTTCACATTCAGCAAACCTGG
*PPARGC1A*	F: CCAAAGGATGCGCTCTCGTTCA
	R: CGGTGTCTGTAGTGGCTTGACT

**Table 2:** Concentrations of identified phenolic compounds (mg/100 g sample DW) in Tombul and Palaz hazelnut extracts identified by HPLC.

	Tombul	Palaz
Gallic Acid	0.12 ± 0.02	0.04 ± 0.03
Protocatechulic Acid	5.18 ± 0.02	4.05 ± 0.01
Vanillic Acid	1.97 ± 0.02	1.92 ± 0.01
Catechin	1.86 ± 0.23	1.67 ± 0.03
Epigallocatechin Gallate	1.52 ± 0.23	0.62 ± 0.01
Epicatechin	9.89 ± 0.75	8.28 ± 0.11
Syringic Acid	0.59 ± 0.00	0.51 ± 0.02
t-cinnamic acid	0.07 ± 0.01	0.07 ± 0.01
p-coumaric acid	0.24 ± 0.00	0.09 ± 0.00

Data were expressed as the mean ± SD (n = 3).

**Table 3:** mRNA gene expression fold changes in *MICU1*, *MICU2, PPAR-γ, PPARGC1A, UCP1, UCP2* and *UCP3* genes after exposure to hazelnut extract as compared to control.

Target Gene	Fold Change (Palaz Extract)	*p* Value	Fold Change (Tombul Extract)	*p* Value
*MICU1*	+129.5 ± 17.71	<0.001	+163.99 ± 13.65	<0.001
*MICU2*	+149.0 ± 16.77	<0.001	+136.75 ± 0.03	<0.001
*UCP1*	−0.07 ± 0.007	<0.001	−0.11 ± 0.02	<0.001
*UCP2*	+14.73 ± 1.95	<0.001	+31.34 ± 0.61	<0.001
*UCP3*	+20.08 ± 2.94	<0.05	+13.88 ± 0.47	<0.001
*PPAR-γ*	+3.78 ± 0.07	<0.001	+7.42 ± 1.80	<0.05
*PPARGC1A*	0.81 ± 0.07	0.062	1.22 ± 0.33	0.44

## Data Availability

The original contributions presented in this study are included in the article. Further inquiries can be directed to the corresponding author.

## Abbreviations

The following abbreviations are used in this manuscript:

CRC	Colorectal cancer
GAE	Gallic acid equivalent
GAPDH	glyceraldehyde-3-phosphate dehydrogenase
HPLC	High-Performance Liquid Chromatography
IC_50_	Inhibitory Concentration
MICU1	Mitochondrial calcium uptake 1
MICU2	Mitochondrial Calcium Uptake 2
MMP	Mitochondrial Membrane Potential
PDA	Photodiode Array
PPARγ	Peroxisome proliferator-activated receptor gamma
PPARGC1A	Peroxisome proliferator-activated receptor gamma coactivator 1 alpha
RT	Retention Time
UCP1	Uncoupling Protein 1
UCP2	Uncoupling Protein 2
UCP3	Uncoupling Protein 3
